# Neural mechanisms underlying the Rubber Hand Illusion: A systematic review of related neurophysiological studies

**DOI:** 10.1002/brb3.2124

**Published:** 2021-07-21

**Authors:** Stefan Golaszewski, Vanessa Frey, Aljoscha Thomschewski, Luca Sebastianelli, Viviana Versace, Leopold Saltuari, Eugen Trinka, Raffaele Nardone

**Affiliations:** ^1^ Department of Neurology Christian Doppler Klinik Paracelsus Medical University Salzburg Austria; ^2^ Karl Landsteiner Institut für Neurorehabilitation und Raumfahrtneurologie Salzburg Austria; ^3^ Spinal Cord Injury and Tissue Regeneration Center Salzburg Austria; ^4^ Department of Neurorehabilitation Hospital of Vipiteno (SABES‐ASDAA) Vipiteno‐Sterzing Italy; ^5^ Research Unit for Neurorehabilitation South Tyrol Bolzano Italy; ^6^ Centre for Cognitive Neuroscience Salzburg Salzburg Austria; ^7^ University for Medical Informatics and Health Technology UMIT Hall in Tirol Austria; ^8^ Department of Neurology Hospital of Merano (SABES‐ASDAA) Merano‐Meran Italy

**Keywords:** electroencephalography, event‐related potentials, evoked potentials, rubber hand illusion, transcranial magnetic stimulation

## Abstract

**Introduction:**

Many researchers took advantage of the well‐established rubber hand illusion (RHI) paradigm to explore the link between the sense of body ownership and the different brain structures and networks. Here, we aimed to review the studies that have investigated this phenomenon by means of neurophysiological techniques.

**Methods:**

The MEDLINE, accessed by Pubmed and EMBASE electronic databases, was searched using the medical subject headings: “Rubber hand illusion” AND “Transcranial magnetic stimulation (TMS)” OR “Evoked potentials (EP)” OR “Event related potentials (ERP)” OR “Electroencephalography (EEG)”.

**Results:**

Transcranial magnetic stimulation studies revealed a significant excitability drop in primary motor cortex hand circuits accompanying the disembodiment of the real hand during the RHI experience and that the perceived ownership over the rubber hand is associated with normal parietal–motor communication. Moreover, TMS provided causal evidence that the extrastriate body area is involved in the RHI and subsequently in body representation, while neuromodulation of ventral premotor area and the inferior parietal lobe did not result in an enhancement of embodiment. EP and ERP studies suggest that pre‐existing body representations may affect larger stages of tactile processing and support predictive coding models of the functional architecture of multisensory integration in bodily perceptual experience. High‐frequency oscillations on EEG play a role in the integrative processing of stimuli across modalities, and EEG activity in γ band activity in the parietal area reflects the visuotactile integration process. EEG studies also revealed that RHI is associated with the neural circuits underlying motor control and that premotor areas play a crucial role in mediating illusory body ownership.

**Conclusion:**

Neurophysiological studies shed new light on our understanding of the different aspects that contribute to the formation of a coherent self‐awareness in humans.

## Graphical abstract

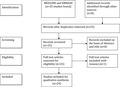

## 1 INTRODUCTION

The rubber hand illusion (RHI) paradigm experimentally produces an illusion of rubber hand ownership and arm shift by simultaneously stroking a rubber hand in view and a participant's visually occluded hand (Botvinick & Cohen, 1998). Since during RHI the subjects perceive a fake hand as part of their own body, this paradigm represents therefore a well‐known experimental manipulation of body ownership and has been used to induce an illusory feeling of owning the dummy hand through congruent multisensory, visuotactile stimulation. RHI refers to the ability to recognize our body as our own, which allows us to interact properly with the outside world.

The dummy hand is incorporated in the mental representation of one's body through a multisensory integration mechanism.

The conscious experience of being the author of our own actions is thought to be grounded in prereflective and low‐level sensorimotor representations of the self as different from the other.

Therefore, the RHI paradigm also enables to investigate how the brain resolves conflicting multisensory evidence during perceptual inference and can grant insights into how our brain represents our body as our own.

We aimed here at identifying the neurophysiological studies currently available in the literature that have examined this phenomenon. Transcranial magnetic stimulation (TMS), evoked potentials (EP), event‐related potentials (ERP), and electroencephalography (EEG) studies were reviewed and discussed.

## 2 METHODS

A literature review was conducted using MEDLINE, accessed by Pubmed (1966–June 2020) and EMBASE (1980–June 2020) electronic databases. The following medical subject headings (MeSH) and free terms were searched: “rubber hand illusion (RHI)” AND “transcranial magnetic stimulation (TMS)” OR “repetitive transcranial magnetic stimulation (rTMS)” OR “evoked potentials (EP)” OR “event related potentials” (ERP) OR “electroencephalography (EEG)”. Original articles written in English were considered eligible for inclusion, while review articles and single case reports were excluded. Techniques that reproduce the well‐known rubber hand illusion, but in virtual reality, were excluded.

For the selected titles, full‐text articles were retrieved, and reference lists of them were searched for additional publications. The principal investigators of included studied were contacted when necessary to require additional information. Two review authors independently screened the titles and abstracts of the initially identified studies and then assessed the methodological quality of each study and risk of bias, including blinding. This search strategy yielded 24 results (13 TMS, 4 EP or ERP, 7 EEG studies).

**FIGURE 1:**
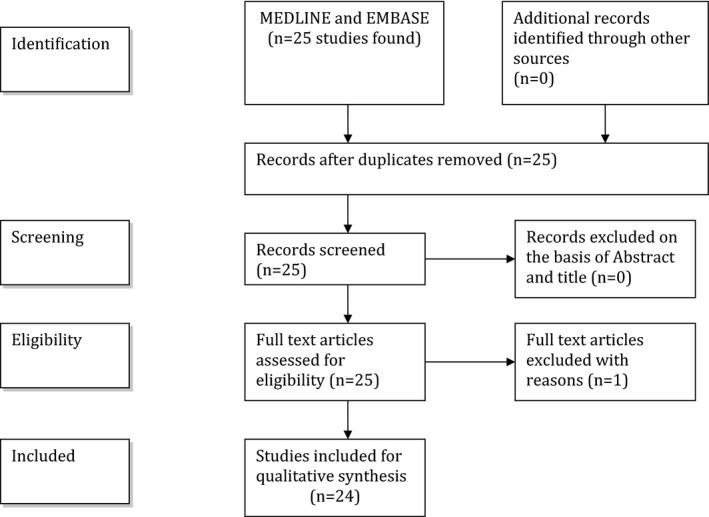
Flowchart showing the selection/inclusion process

A flowchart (Figure 1) illustrates the selection/inclusion process.

The methodological and technical aspects of the reviewed TMS, EEG, and SEP studies are illustrated in the Tables 1 and 2.

**TABLE 1:** Methodological and technical aspects of the TMS studies

Study	*n*	Age	TMS paradigms.	*F* (Hz)	Intensity % of rMT	Time	Site	Neuronav.
Wold et al. (2014)	16		rTMS	1	80/40	20 min	Left EBA	Yes
Peviani et al. (2018)	24	23.9	rTMS	1	100	15 min	Right PMv	Yes
Kammers et al. (2009)	13	22.2	rTMS	1	80	20 min	Left IPL	*ex post
Fossataro et al. (2018)	32	24	rTMS	1	90	20 min	Hand area left M1	No
			MEP		100		Hand area right M1	No
Frey et al. (2020)	18	30.9	iTBS		80		Right S1	No
Mioli et al. (2018)	25		iTBS		80		Right PMv/right IPL	Yes
Ticini et al. (2018)	16	26.4	imTBS/cTBS		80		Left IPL	No
			MEP		120		Left M1	No
Della Gatta et al. (2016)	44		MEP		110		Hand area left M1	No
Schütz‐Bosbach et al. (2006)	14	24	MEP		105		Hand area left M1	No
Schütz‐Bosbach et al. (2009)	15	24.7	CSP		**130		Hand area left M1	No
Burin et al. (2017)	14		MEP/rMT		120		Hand area left/right M1	No
Karabanov et al. (2017)	7	31.9	MEP		100		Hand area right M1	Yes
	20		MEP/ISI/pp		100		Right M1/right aIPS	Yes
Isayama et al. (2019)	26	45.2	MEP/pp		90		Left PPC‐left M1	Yes
	27	44.7	SAI/LAI/DNS				Hand area left M1	No

Abbreviation: aIPS, Anterior intraparietal sulcus; CSP, Cortical silent period; cTBS, Continuous theta burst stimulation; DNS, Digital nerve stimulation; EBA, Extrastriate body area; *F*, frequency; imTBS, Intermediate theta burst stimulation; IPL, Inferior parietal lobule; ISI, Inter stimulus interval; iTBS, Intermittent theta burst; LAI, Long‐latency afferent inhibition; M1, Primary motor cortex; MEP, Motor‐evoked potential; MRI, Magnetic resonance imaging; PMv, Ventral premotor cortex stimulation; pp, paired pulse; rMT, Resting motor threshold; rTMS, Repetitive transcranial magnetic stimulation; S1, Primary somatosensory cortex; SAI, Short‐latency afferent inhibition.

**TABLE 2:** Methodological and technical aspects of the EEG and SEP studies

Study	*n*	Age	EEG	EEG	SEP	Freq. (Hz)	EEG analysis
			*n* channels	Montage			
Kanayama et al. (2007)	11	19–22	15	Linked mastoid reference	Yes (stimulus‐based)	1–100	ERP peak amplitude and phase synchrony analysis
Kanayama et al. (2009)	24	18–24	32	Single reference (nose tip)	Yes (stimulus‐based)	1–100	ERP peak amplitude, time–frequency, and phase synchrony analysis
Kanayama et al. (2017)	20	19–36	64	Average reference	No	1–125	Causality analysis via renormalized partial directed coherence on cluster‐ based dependent EEG components
Faivre et al. (2017)	23	Mean 22.4	64	Average reference	No	1–45	Band‐specific power analysis, source localization, and phase synchrony calculation in source space
Rao and Kayser (2017)	20	Mean 22.6	64	Average reference	Yes (stimulus‐based)	0.5–30	Average ERP and spectral power analyses
Shibuya et al. (2018)	18	Mean 23.6	32	Linked mastoid reference	No	8–13	Time–frequency analysis via event‐related spectral perturbation
Zeller et al. (2015)	13	21–32	64	Average reference	Yes (brush‐based)	2–20	ERP activation map comparisons within sensor and source space
Zeller et al. (2016)	13	21–32	64	Average reference	Yes (brush‐based)	2–20	Dynamic causal modeling of the SSEP response in source space
Peled et al. (2003)	38	Mean 28.5	11	Average reference	Yes (brush‐based)	1–70	Amplitude–latency analyses of ERP response
Press et al. (2008)	32	20–42	23	Linked mastoid reference	Yes (stimulus‐based)	0.1–40	ERP amplitude analyses

Abbreviations: EEG, electroencephalography; ERP, event‐related potential; Hz, Hertz; *n*, number; SEP, somatosensory evoked potentials.

## 3 TRANSCRANIAL MAGNETIC STIMULATION

### 3.1 Motor cortex

Transcranial magnetic stimulation (TMS) is an approach that, if delivered repetitively, can influence brain function. Repetitive TMS (rTMS) can be applied as continuous trains of low frequency (LF) or bursts of higher‐frequency (HF) rTMS. Generally, LF rTMS (stimulus rates ≤1 Hz) induces inhibitory effects on motor cortical excitability, whereas HF rTMS (≥3 Hz) usually promotes an increase in cortical excitability (Fitzgerald et al., 2006; Lefaucheur, 2019).

A coherent self‐awareness implies the existence of a tight link between the sense of body‐ownership and the motor system. To assess the effects of the motor system down‐regulation on the RHI susceptibility, in a sham‐controlled study the primary motor cortex (M1) excitability was modulated by off‐line LF rTMS.

Participants underwent the RHI after real or sham rTMS, either on the right hand, contralateral to the inhibited hemisphere or on the left hand in the Experiment 1, ipsilateral to the inhibited hemisphere, in Experiment 2 (Fossataro et al., 2018).

Subjective and objective RHI measures, that are the Embodiment/Disembodiment Questionnaires and the Proprioceptive Drift (PPD), respectively, were assessed. Only in Experiment 1, rTMS strengthened the illusory experience, as revealed by a significant increase of both measures in the real compared to sham group. This finding demonstrates that the down‐regulation of the M1 activity can lead to an attenuation of the sense of body ownership, so that the subject becomes more prone to incorporate an alien body part. This evidence also suggests the presence of a mutual interaction between the sense of body ownership and the motor system, shedding new light on the construction mechanisms of a coherent sense of self as an acting body.

**FIGURE 2:**
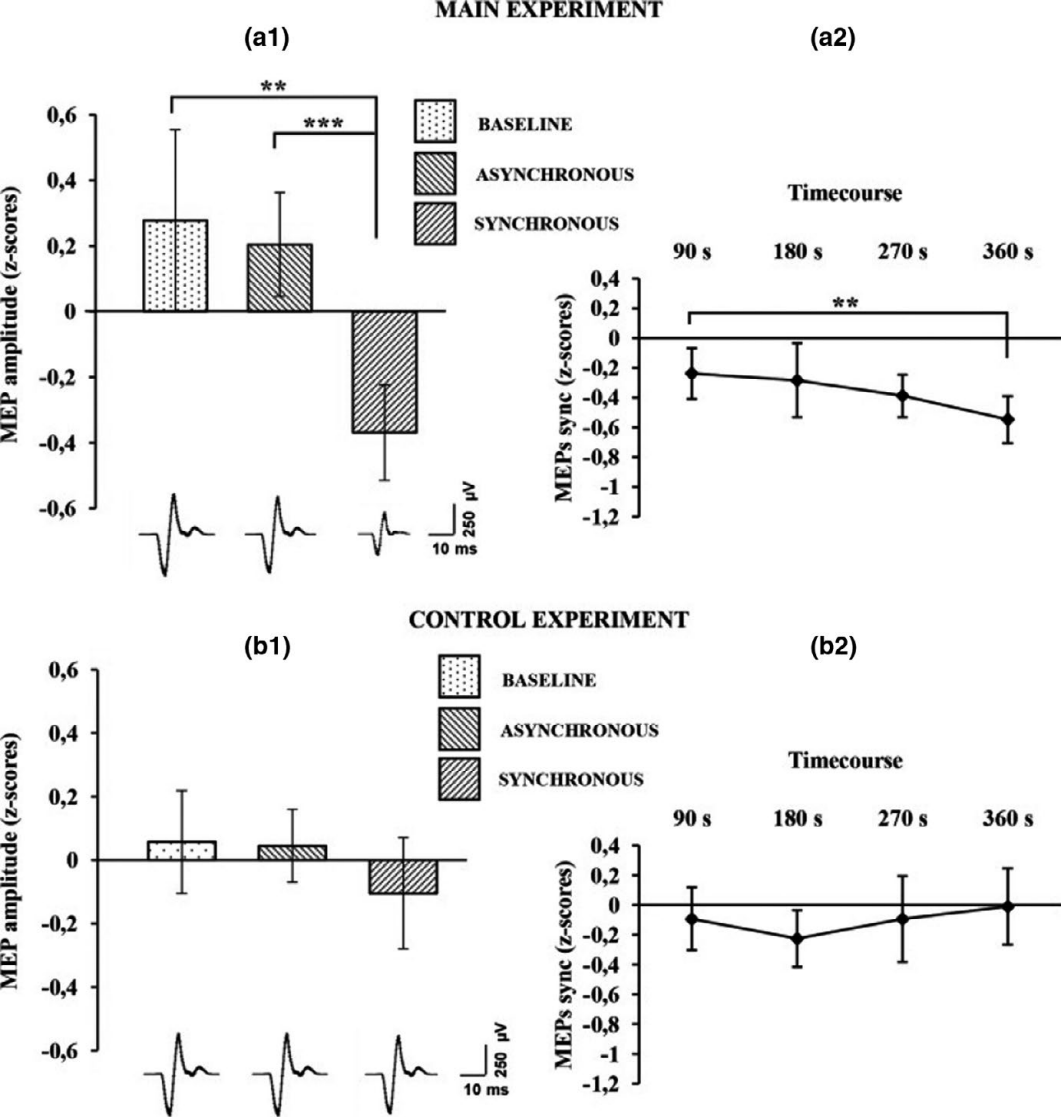
Average motor‐evoked potential (MEP) amplitude variation in the first‐dorsal interosseus (FDI) muscle recorded across all subjects is plotted in a_1_, for the main experiment, and in b_1_ for the control experiments. Histograms represent the peak‐to‐peak MEP mean amplitude (normalized) ±95% CI in the baseline, asynchronous, and synchronous conditions, respectively. Significant levels: **p < .005; ***p < .0001. Average MEP amplitude profile recorded across all subjects in the synchronous condition are plotted in a_2_ for the main experiment and in b_2_ for the control experiment; points represent the peak‐to‐peak MEP mean amplitude (normalized), ±95% CI, at four time points after induction of the illusion (90, 180, 270 and 360 s); significance level: **p < .005. Examples of average raw MEPs recorded from two representative subjects (for the main and control experiments) in the baseline (main: 609 µVolt; control: 619 µVolt), asynchronous (main: 771 µVolt; control: 601 µVolt), and synchronous (main:150 µVolt; control: 583 µVolt) conditions. Reproduced with permission from Della Gatta et al. (2016)

During the RHI, subjects experience an artificial hand as part of their own body, while the real hand is subject to a sort of “disembodiment.” To investigate whether this altered belief about the body also affect physiological mechanisms involved in body ownership, such as motor control, the effect of this illusion on the excitability of the motor pathways to the real (disembodied) hand has been assessed (Della Gatta et al., 2016). A significantly reduced motor‐evoked potentials (MEPs) amplitude from the real hand was found, with respect to baseline, when subjects in the synchronous, but not in the asynchronous, condition experience the fake hand as their own (Figure 2).

### 3.2 Motor system—Observation of action and action

Observation of another's action can selectively facilitate the brain's motor circuits for making the same action. A "mirror‐matching mechanism" might map observed actions onto the observer's own motor representations. This view implies that the brain represents others' actions like one's own. However, the different experience of one's own body from that of others' bodies with regard to viewpoint, morphological features, familiarity, and the hallmark feature of kinesthetic experience makes this hypothesis difficult to test.

**FIGURE 3:**
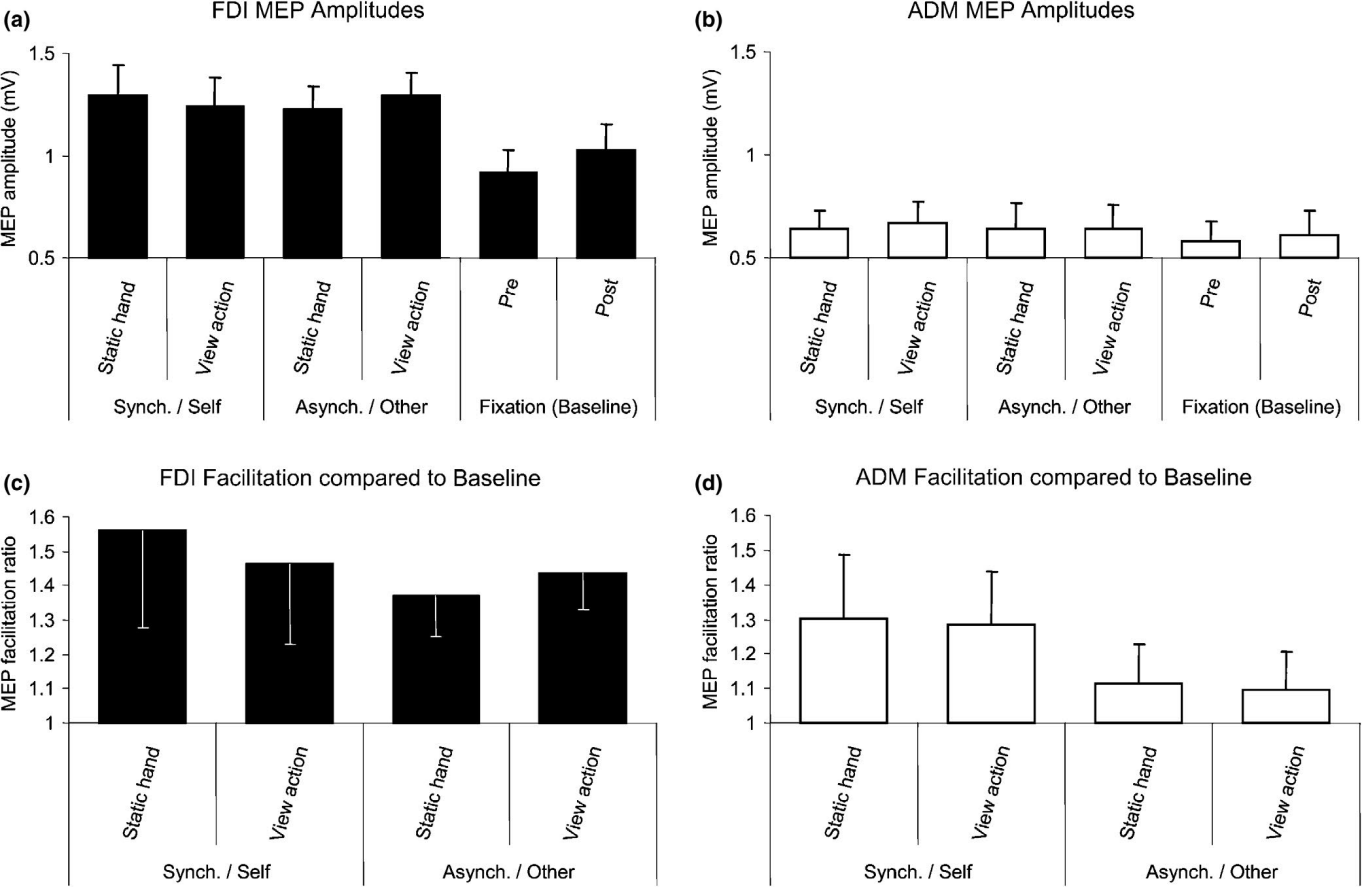
Mean + Standard Error, SE, Cortical Excitability of FDI and ADM after Action Observation or during Viewing of a Static Hand in Each Ownership Condition (a and b) Average MEP size in each condition. (c and d) Facilitation ratios in the experimental blocks relative to averaged baseline blocks

Schütz‐Bosbach used RHI to compare effects of observing actions that either were or not illusorily attributed to the subject's own body, and TMS to directly compare action facilitation effects produced by observing an action attributed to another's body with the facilitation effects produced by observing actions of body that appears to be one's own (Schütz‐Bosbach et al., 2006). The authors found that watching another's actions facilitated the motor system, while observing identical actions, which were illusorily attributed to the subject's own body, displayed the opposite pattern (Figure 3). Therefore, motor facilitation strongly depends on the agent to whom the observed action is attributed. In contrast to previous concepts of equivalence between one's own actions and actions of others, these results suggest that social differentiation, rather than equivalence, is characteristic of the human action system.

Many studies indicate that both self‐generated actions and observation of others’ actions activate overlapping neural networks, implying a shared, agent‐neutral representation of self and other. Contrary to the shared representation hypothesis, it has been demonstrated that the human motor system is not neutral with respect to the agent of an observed action (Schütz‐Bosbach et al., 2009). Unlike the observation of identical actions linked to the self, observation of actions attributed to another agent facilitated the motor system. The same research group also investigated whether the absence of motor facilitation for observing one's own actions may reflect cortical inhibitory processes related to self‐representation. The duration of the cortical silent period (CSP) induced by TMS applied over M1 in active muscles reflects the activity of intracortical inhibitory GABAergic circuits (Paulus et al., 2008) and can thus be considered as an indicator of motor cortex inhibition. Using the manipulation of body ownership based on the RHI, the authors found that observation of actions linked to the self led to longer CSP than observation of a static hand, while observing identical actions attributed to another agent led to the opposite effect. This finding probably reflects an inhibition of the motor system associated with self‐representation. The suppression at cortical level for actions linked to the self might be useful to avoid inappropriate perseveration within the motor system.

There is uncertainty about whether, and to what extent, actions contribute to constructing awareness of one's own body. The aim of another study was to investigate, at both physiological and behavioral level, the effects of a prolonged limb immobilization on body ownership (Burin et al., 2017). A group of healthy participants, whose left‐hand movements were prevented by a cast for 1 week, and a control group without any movement restriction, were examined. In both groups, the strength of the RHI (in particular PPD and questionnaire on ownership) and some physiological parameters which are known to be modulated by short‐term arm immobilization, such as MEPs amplitudes, resting motor threshold (RMT), and force parameters, were assessed before and after the week of immobilization. The results showed stronger illusory effects on the immobilized hand on both behavioral indexes and weaker illusory effects on the nonimmobilized hand on the questionnaire. Additionally, the increased PPD was positively correlated with the RMT of the contralateral hemisphere. These findings demonstrated that the alteration of those movement‐related signals, which continuously originate from our own body parts, can modulate the experience of those body parts as mine. This, in turn, supports the view that actions play a direct role in the developing and maintaining a coherent body ownership.

### 3.3 Parietal cortex

Neuroimaging data demonstrated an involvement of the posterior parietal cortex (PPC) when recalibration of the perceived position of the participant's real hand toward the rubber hand occurs. Off‐line LF rTMS in a double‐blind, sham‐controlled with subjects design was used to explore the role of the inferior posterior parietal lobule (IPL) in inducing the RHI directly (Kammers et al., 2009). rTMS over the IPL attenuated the strength of the RHI for immediate perceptual body judgments only, while delayed perceptual responses were unaffected. Also ballistic action responses and subjective self‐reports of feeling of ownership over the rubber hand remained unaffected after rTMS over the IPL. These findings are consistent those of with previous studies indicating that the illusion can be broken down into dissociable bodily sensations. RHI does not only affect the embodiment of the rubber hand but also independently influences the experience and localization of one's own hand in an independent manner. These findings are also in agreement with a multicomponent model of somatosensory body representations, wherein the IPL plays a crucial role in establishing perceptual body judgments, but not actions or higher‐order affective bodily judgments.

It has been hypothesized that the IPL is generally involved in self‐other differentiation processes and in providing an explicit sense of action authorship.

A protocol of rTMS named theta burst stimulation (TBS) employs low intensities and has a robust, long‐lasting effect in normal subjects (Di Lazzaro et al., 2005; Huang et al., 2005). Continuous TBS (cTBS) decreases cortical excitability, while intermittent TBS (iTBS) was shown to increase motor cortical excitability.

**FIGURE 4:**
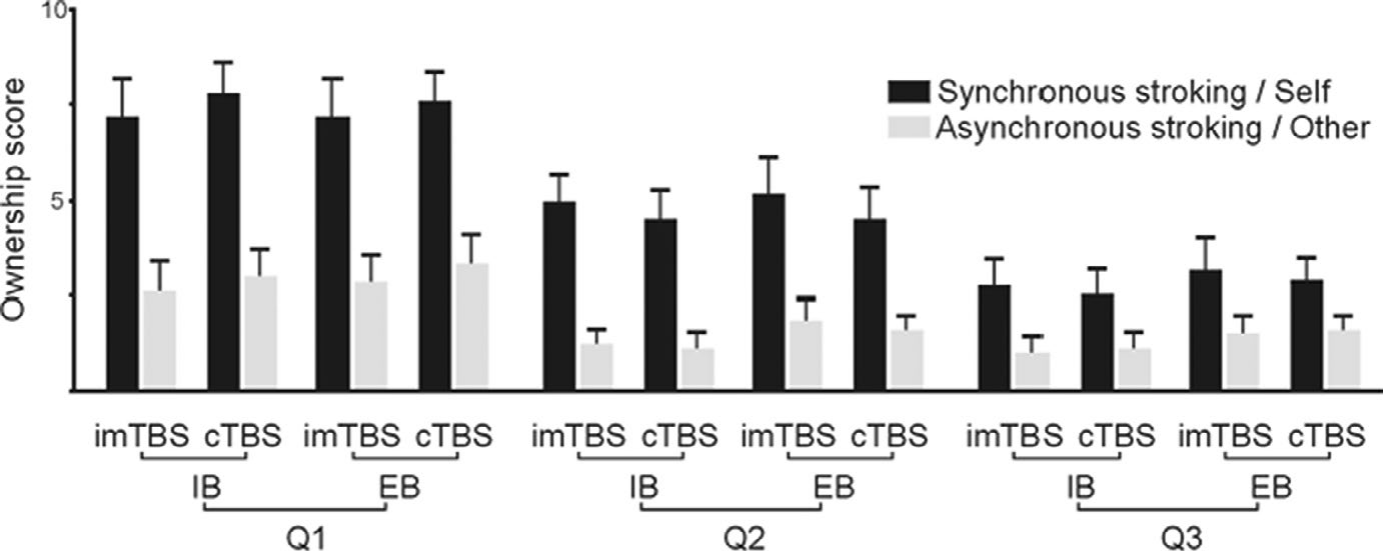
Means and standard errors of the RHI questionnaire assessing the ownership of the observed hand in the different experimental conditions: The conditions were the following: TBS (cTBS and imTBS), BLOCK (IB and EB) and STROKING (Synchronous/Self and Asynchronous/Other). Legend: TBS = theta burst stimulation; cTBS = continuous theta burst stimulation; imTBS = intermediate theta burst stimulation; IB = Induction Block; EB = Experimental Block. Reproduced with permission from Ticini et al. (2018)

To provide further evidence for the causal and functional role of IPL in distinguishing self‐related and other‐related sensorimotor representations, another study employed TBS to condition left IPL's activity before a social version of the RHI led participants to illusorily attribute observed finger movements to their own body (Ticini et al., 2018). MEPs to single‐pulse TMS over the M1 as proxies of action authorship during action observation have been recorded. In a control condition (intermediate TBS over the left IPL), others' actions facilitated whereas self‐attributed movements inhibited the motor system, while cTBS disrupted this mismatch between self and other representations (Figure 4). This finding supports the fundamental role of the IPL's role in providing important authorship signals for social differentiation in the human action system.

### 3.4 Ventral premotor area

Great attention has been devoted to the neural functional correlates of RHI mechanisms highlighting the pivotal role of an occipito‐parieto‐frontal network involving the ventral premotor area (PMv). The specific role of the PMv in generating the sense of ownership is still poorly understood. A study aimed at exploring the role of PMv in generating and experiencing the RHI (Peviani et al., 2018). Off‐line rTMS was delivered to a group of 24 healthy participants, while changes in proprioceptive judgment and self‐reported illusion sensations were collected and analyzed separately. The PMv was found not to be directly implicated in generating the sense of ownership. In fact, its inhibition affected the explicit detection of the visuotactile congruence without interfering with the illusion experience itself. It has been hypothesized that the conscious visuotactile congruence detection may be independent from the conscious illusion experience. These results also support the notion that the RHI grounds on a complex interaction between bottom‐up and top‐down processes, as the visuotactile integration per se may be not sufficient to trigger the subjective illusion.

An enhanced sense of prosthesis ownership may be the key for higher amputees' quality of life. In 28 healthy subjects, neuronavigated iTBS delivered over the right PMv or IPL has been tested, compared to sham stimulation, to enhance embodiment in the RHI paradigm (Mioli et al., 2018). Neuromodulation of both areas did not result in an enhancement of embodiment, as assessed by the results collected from a self‐evaluation questionnaire for the extent of self‐attribution of the rubber hand and PPD. In all cases, the difference between synchronous and asynchronous stroking confirms the successful induction of the illusion. The low consistency of iTBS over brain regions other than M1 might account for the absence of effect. Therefore, other neuromodulating techniques, acting on cortical networks different from the ones sensitive to iTBS, should be tested to enhance artificial hand embodiment.

### 3.5 Parieto‐frontal connections

Parieto‐frontal connections are thought to play an important role in adjusting body ownership during the RHI. Using a moving RHI paradigm (Kalckert & Ehrsson, 2012), single‐site and dual‐site TMS has been applied to explore corticospinal and parietal–frontal connectivity during perceived rubber hand ownership (Karabanov et al., 2017). Healthy volunteers received a conditioning TMS pulse over left anterior intraparietal sulcus (aIPS) and a test TMS pulse over left M1. MEPs were recorded at rest and during three RHI conditions: agency and ownership, agency but no ownership, neither agency nor ownership. Parietal–motor communication differed among experimental conditions. The induction of action ownership was associated with an inhibitory parietal‐to‐motor connectivity, which was comparable to the aIPS‐to‐M1 inhibition present at rest. This aIPS‐to‐M1 inhibition disappeared during movement conditions not inducing ownership. Corticospinal excitability was not significantly modulated during the motor RHI as indicated by the task‐constant MEP amplitude elicited by the M1 test pulse alone. These results indicate that the perceived ownership over the rubber hand is associated with normal parietal–motor communication, which is disturbed if the sensorimotor conflict between one's own hand and the rubber hand is not resolved.

Rubber hand illusion involves visual, tactile, and proprioceptive multisensory integration and activates different multisensory brain areas, including the PPC. Multisensory inputs are transformed into outputs for motor control in association areas such as PPC. A behavioral study reported decreased motor performance after RHI. However, it is still uncertain whether RHI may change the interactions between sensory and motor systems as well as between PPC and the M1. TMS was used to evaluate the functional connections from the primary somatosensory and association cortices to M1 and from PPC to M1 during RHI (Isayama et al., 2019). In the first experiment, short‐latency afferent inhibition (SAI) and long‐latency afferent inhibition (LAI) were measured before and immediately after a synchronous or an asynchronous condition (RHI and control, respectively). In the second experiment, PPC‐M1 interaction was evaluated using two coils. SAI and LAI were found to be reduced in the synchronous condition compared with baseline, thus suggesting that RHI decreased somatosensory processing in the primary sensory and the association cortices projecting to M1. The authors also reported greater inhibitory PPC‐M1 interaction, which was related to stronger RHI, as assessed by questionnaire. These findings suggest that RHI modulates both the early and late stages of tactile afferents and central processing, which leads to altered excitability by reducing the gain of somatosensory afferents to resolve conflicts among multisensory inputs. Therefore, perception of one's own body parts involves integrating different sensory information and is important for motor control. The reduced effects of cutaneous stimulation on motor cortical excitability during RHI might reflect decreased gain of tactile input to resolve multisensory conflicts. RHI strength correlated with the degree of inhibitory PPC‐M1 interaction, indicating that parietal–motor connections are involved in resolving sensory conflicts and body ownership during RHI.

### 3.6 Other areas

RHI can provide insights into how our brain represents our body as our own. Recent studies have demonstrated an involvement of the extrastriate body area (EBA), an area of the brain that is typically implicated in the perception of nonface body parts, in illusory body ownership. To investigate the possible causal role EBA in the RHI, sixteen healthy subjects took part in a sham‐controlled, 1 Hz rTMS experiment (Wold et al., 2014). Participants received (RHI condition) or asynchronous (control) stroking and were asked to report the perceived location of their real hand, as well as the intensity and the temporal onset of experienced ownership of the dummy hand. Following rTMS of the left EBA, participants misjudged their real hand's location significantly more toward the dummy hand during the RHI than after sham stimulation. This difference in "proprioceptive drift" (PPD) provides the first causal evidence that the EBA is involved in the RHI and subsequently in body representation. Therefore, these results further support the view that the EBA is necessary for multimodal integration.

A recent cross‐over, placebo‐controlled, single‐blind study aimed at assessing whether RHI, in combination with HF rTMS given as iTBS applied over the hand area of the primary sensory region (S1), can enhance tactile sensation in a group of 21 healthy subjects and one patient with cervical spinal cord injury (SCI; Frey et al., 2020). Among the four sessions, which covered all combinations of real and sham stimulations of the RHI and the TBS, the sham TBS and real RHI condition shows the greatest effect on the PPD and on the score of RHI questionnaires in the healthy subjects and in the patient. Conversely, the upregulation of the cortical excitability of S1 via TBS seems to impair the effect of the RHI, probably due to a strengthening of the top‐down connection between the central nervous system and the periphery, diminishing the RHI.

## 4 EVOKED AND EVENT‐RELATED POTENTIALS

The RHI is enhanced in schizophrenia patients. Somatosensory evoked potentials (SEPs) during the illusion were compared between schizophrenia patients and normal control subjects (Peled et al., 2003). While normal subjects showed significant preillusion—illusion differences at early as well as at late SEP components, schizophrenia patients fail to show such alterations. The results of this study are consistent with previous findings pointing to alterations in associative brain regions in schizophrenia.

Press and coworkers investigated how the integration of seen and felt tactile stimulation modulates somatosensory processing; they also studied whether visuotactile integration depends on temporal contiguity of stimulation, and its coherence with a pre‐existing body representation (Press et al., 2008). During the training phases, participants viewed a rubber hand or a rubber object that was tapped either synchronously with stimulation of their own hand, or in an uncorrelated fashion. During the test phases, somatosensory event‐related potentials (ERPs) to tactile stimulation of the left or right hand were recorded, in order to assess how tactile processing was affected by previous visuotactile experience during training. An enhanced somatosensory N140 component was elicited after synchronous, compared with uncorrelated, visuotactile training, irrespective of whether participants viewed a rubber hand or rubber object. This early effect of visuotactile integration on somatosensory processing might represent a potential electro‐physiological correlate of the RHI that is induced by temporal contiguity, but not by pre‐existing body representations. ERP modulations were observed beyond 200 ms poststimulus, suggesting an attentional bias determined by visuotactile training. These late modulations were absent when the stimulation of a rubber hand and the participant's own hand was uncorrelated during training, suggesting that pre‐existing body representations may affect later stages of tactile processing.

In order to identify the functional anatomy of the RHI, multichannel EEG, acquired under three conditions of tactile stimulation, has been used (Zeller et al., 2015). Evoked potentials (EP) were averaged from EEG signals registered to the timing of brushstrokes to the participant's hand. The participant's hand was stroked either in the absence of an artificial hand (REAL) or synchronously with an artificial hand, which either lay in an anatomically plausible (CONGRUENT) or impossible (INCONGRUENT) position. The illusion was reliably induced in the CONGRUENT condition. For right‐hand stimulation, significant differences between conditions emerged at the sensor level around 55 ms after the brushstroke at left frontal and right parietal electrodes. Response amplitudes were smaller for illusory (CONGRUENT) compared with nonillusory (INCONGRUENT and REAL) conditions in the contralateral perirolandic region (pre‐ and postcentral gyri), superior parietal lobule, and IPL, whereas veridical perception of the artificial hand (INCONGRUENT) amplified responses at a scalp region overlying the contralateral postcentral gyrus and IPL compared with the remaining two conditions. Similar contralateral patterns were elicited by the left‐hand stimulation.

Since some studies suggested that the premotor cortex (PMC) is thought to be a pivotal area in RHI, to explore the effective connectivity between—and within—sensory and premotor areas involved in bodily perceptions, a dynamic causal modeling of touch‐evoked responses has been used by the same research group in 13 healthy subjects (Zeller et al., 2016). Also in this study, each subject's right hand was stroked while viewing their own hand ("REAL"), or an artificial hand presented in an anatomically plausible ("CONGRUENT") or implausible ("INCONGRUENT") position. Bayesian model comparison revealed strong evidence for a differential involvement of the PMC in the generation of touch‐evoked responses under the three conditions, providing further support that there being a crucial role of PMC in bodily self‐attribution. Indeed, the extrinsic (forward) connection from left occipital cortex to left PMC was stronger for CONGRUENT and INCONGRUENT as compared to REAL, reflecting the augmentation of bottom‐up visual input when multisensory integration is very difficult. The intrinsic connectivity in the primary somatosensory cortex (S1) was attenuated during the illusory percept in the CONGRUENT condition.

These findings of these EP studies support predictive coding models of the functional architecture of multisensory integration (and attenuation) in bodily perceptual experience.

## 5 ELECTROENCEPHALOGRAPHY

**FIGURE 5:**
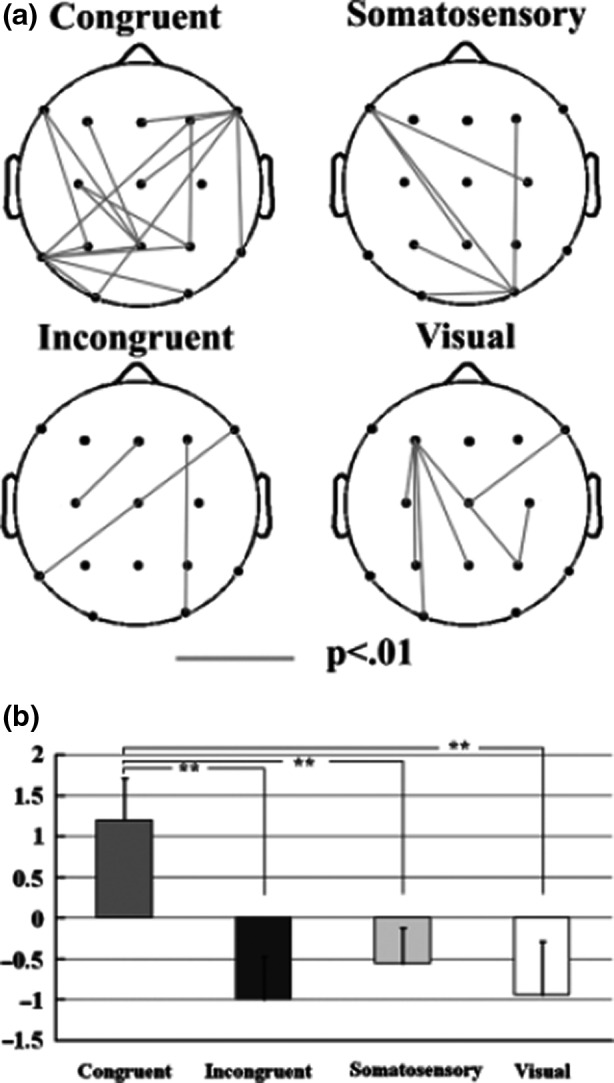
A: The phase synchrony between electrode sites for the congruent and incongruent conditions. The lines denote the significant synchrony between connected electrodes (p<.01). B: The bar graph for the averaged phase‐locking values across all pairs of electrodes. **p<.001. bar graph

The integration of multimodal stimuli is thought to be important for the promotion of adaptive behavior. Several studies have identified brain areas that respond to multimodal stimuli, but the temporal features are not been clarified. Initial ERP studies have demonstrated crossmodal attention effects, but the exact mechanisms underlying crossmodal integration have not been clarified. Electroencephalography (EEG) activity in the γ‐band can be considered as a correlate of multimodal integration. In the first study of Kanayama and colleagues, participants localized a tactile stimulus on their fingers while seeing visual stimuli on rubber hands with the same posture as their hands (Kanayama et al., 2007). EEG analyses using wavelet transform indicated there is relationship between that interelectrode phase synchrony in the gamma‐band range (40–50 Hz) and behavioral indices of the intermodal illusion under consideration (Figure 5). The findings suggest a role of high‐frequency oscillations in the integrative processing of stimuli across modalities.

In a subsequent study, the effect of the RHI on the crossmodal integration process by measuring EEG has been investigated (Kanayama et al., 2009). The participants who experienced less intensive illusion showed greater congruency effect on reaction time (RT), greater power increase at the parietal zero electrode (Pz), and smaller interelectrode synchrony of the γ‐band activity. On the other hand, the subjects who experienced more intense illusion showed greater interelectrode synchrony. These findings suggested that the γ‐band activity in the parietal area reflects the visuotactile integration process and that its synchrony causes the illusory intensity.

Since RHI is linked to a frontoparietal circuit, another study aimed at exploring the associated dynamics of neural oscillation (Kanayama et al., 2017). EEG was recorded while delivering spatially congruent/incongruent visuotactile stimulations to fake and real hands. Time–frequency analyses and calculated renormalized partial directed coherence (rPDC) were applied to examine cortical dynamics during the RHI. When visuotactile stimulation was spatially congruent, and the fake and real hands were aligned, a reduced causal relationship from the medial frontal to the parietal regions with respect to baseline, around 200 ms poststimulus, has been observed. This change in rPDC was negatively correlated with a subjective report of the RHI intensity. Moreover, a link has been observed between the PPD and an increased causal relationship from the parietal cortex (PC) to the right S1 during a relatively late period (550–750 ms poststimulus). These findings suggest a two‐stage process: a reduced influence from the medial frontal regions over the PC unlocks the mechanisms that preserve body integrity, allowing RHI to emerge; the information processed at the PC is back‐projected to the S1 contralateral to the real hand, inducing the PPD.

Vision is thought to be shaped by environmental and bodily signals. In the Taylor illusion, the size of an afterimage projected on one's hand changes according to proprioceptive signals conveying hand position. Faivre and colleagues aimed at assessing whether the Taylor illusion depend, besides the physical hand position, also on bodily self‐consciousness as quantified through illusory hand ownership (Faivre et al., 2017). An afterimage projected on the participant's hand drifted depending on illusory ownership between the participants' two hands, showing an implication of self‐representation during the Taylor illusion, has been detected. Oscillatory power analysis of EEG signals showed that illusory hand ownership was stronger in participants with stronger α suppression over left sensorimotor cortex, whereas the Taylor illusion correlated with higher β/γ power over frontotemporal regions. A higher γ connectivity between left sensorimotor and inferior PC was also found during illusory hand ownership. These findings revealed that afterimage drifts in the Taylor illusion do not only depend on the physical hand position but also on subjective ownership, which itself is based on the synchrony of somatosensory signals from the two hands. The effect of ownership on afterimage drifts is associated with β/γ power and γ connectivity between frontoparietal regions and the visual cortex.

Alteration of body image induced by visuotactile integration is known to be closely related to the activation of the PC, a sensory association area. The expression of brain‐derived neurotrophic factor (BDNF) in the parietal area of macaque monkeys is thought to modulate its activation and alter the extension of body image during tool‐use learning. To clarify the relationship between PC activation related to body image alterations and BDNF levels in humans, the relationship between human serum BDNF levels and EEG responses has been investigated during a visuotactile integration task involving a rubber hand (Hiramoto et al., 2017). Cortical oscillatory components in the high‐frequency (γ) band in the left PC were found.

Furthermore, the power values of these oscillations were positively correlated with serum BDNF levels. Neuroimaging studies have revealed a variety of neurophysiological correlates of illusory hand ownership, with conflicting results likely originating from differences in experimental parameters and control conditions. These limitations were overcome by using a fully automated and precisely timed visuotactile stimulation setup to record evoked responses and oscillatory responses in subjects who felt the RHI (Rao & Kayser, 2017). The authors relied on a combination of experimental conditions to rule out confounds of attention, body‐stimulus position and stimulus duration and on the combination of two control conditions to identify neurophysiological correlates of illusory hand ownership. In two separate experiments, a consistent illusion‐related attenuation of ERPs around 330 ms over frontocentral electrodes, as well as decreases of frontal alpha and beta power during the illusion that could not be attributed to changes in attention, body‐stimulus position or stimulus duration, were observed. These results reveal neural correlates of illusory hand ownership in late and likely higher order rather than early sensory processes, and support a role of premotor and possibly intraparietal areas in mediating illusory body ownership.

Several RHI studies have reported that visual manipulation of the embodied fake hand inversely affects the perceptual processing of the observer's own hand (e.g., thermal or pain sensitivity). A recent study aimed at examining whether motor manipulation of the fake hand similarly affects the observer's motor system (Shibuya et al., 2018). This study employed a novel RHI paradigm wherein stroking was interrupted by unexpected movement of the fake hand (i.e., finger spreading) while measuring EEG. Participants often spontaneously moved their hands in accordance with the movement of the fake hand only in the RHI (synchronous) sessions. EEG analyses revealed enhanced neural activation (mu‐rhythm desynchronization) of the motor system during observation of the fake hand movement. Moreover, motor activation was greater in the synchronous than in the asynchronous condition and significantly correlated with the feeling of body ownership over the fake hand.

## 6 DISCUSSION

Embodied cognition, including the hand ownership, has been widely studied in recent years and has been especially investigated through the RHI.

RHI represents an illusory experience during the mislocalization of own hand when correlated visuotactile stimuli are presented to the actual and fake hands. The spatial and temporal contingencies of visual inputs near a fake hand and physical touches to the real hand are thought to promote this illusion. The visuotactile integration processes appear to cause this illusion, and the corresponding brain activity has been revealed in many studies. Most of the research measured the brain activities during the RHI by using fMRI and neurophysiological techniques. This review highlighted the crucial importance of TMS, EP, ERP, and EEG in exploring the neural basis of RHI.

Rubber hand illusion has proven an important phenomenon for the investigation of body ownership and self/other distinction and allows insights into how the brain resolves conflicting multisensory information regarding body position and ownership.

Several studies successfully modulated the RHI susceptibility by employing LF (1 Hz) rTMS over different brain areas. Two studies reported a significant RHI modulation only in the PPD, but in the opposite direction, depending on the stimulated area: while rTMS over the IPL attenuated the perceived shift toward the fake hand (Kammers et al., 2009), the stimulation of the EBA increased it (Wold et al., 2014).

A “virtual lesion” applied over M1 (Siebner & Rothwell, 2003) strengthens the RHI susceptibility in our subjects, as proved by the significant increase, in rTMS compared to sham stimulation, of subjective and objective RHI measures (Fossataro et al., 2018). These results support the evidence of a relationship between the motor system and the sense of body ownership. These findings are also consistent with previous studies showing a relationship between the motor system and the sense of body ownership. Indeed, during mechanical limb immobilization, the sense of body ownership for the immobilized hand was found to be weaker/more flexible (Burin et al., 2017), and the immobilization procedure in healthy subjects (Avanzino et al., 2011), as well as the constraint‐induced movement therapy (CIMT) in brain‐damaged patients (Wittenberg & Schaechter, 2009), showed increased activity of the hemisphere ipsilateral to the immobilized limb. Furthermore, brain‐damaged patients with a pure form of hemiplegia (Burin et al., 2015), spinal cord injury (Scandola et al., 2014; Tidoni et al., 2014), and focal hand dystonia (Fiorio et al., 2011) may have an altered sense of body ownership, according to their greater susceptibility to the RHI paradigm.

The TMS finding of a reduced MEP amplitude recorded from the real hand (Della Gatta et al., 2016) contributes to the theoretical understanding of the relationship between body‐ownership and motor system and provides physiological evidence that a significant drop in motor excitability in M1 hand circuits accompanies the disembodiment of the real hand during the RHI experience. Future studies may be useful to investigate the presence or absence of linear correlations between MEP amplitude and behavioral measures, including both embodiment and disembodiment of the rubber hand, possibly comparing the physiological parameters of responder and nonresponder subjects.

Conversely, the PMv was found not to be directly implicated in generating the sense of ownership (Peviani et al., 2018). This finding also supports the view that the RHI depends on a complex interaction between bottom‐up and top‐down processes, as the visuotactile integration per se may be not sufficient to trigger the subjective illusion. These results are in agreement with those of Longo and colleagues who have previously demonstrated a dissociation between the strength of the illusion and the visuotactile congruence, as measured by the RHI questionnaire (Longo et al., 2008).

The parietal‐to‐motor connectivity pattern observed at rest is also preserved during RHI, thus suggesting the result of a successful resolution of a sensory–motor conflict rather that the illusion of ownership is not an effortful construct. Therefore, perceived ownership modulates changes in aIPS‐M1 connectivity, but does not affect corticospinal excitability directly (Karabanov et al., 2017).

Contrary to the shared representation hypothesis, it has been demonstrated that the human motor system is not neutral with respect to the agent of an observed action (Schütz‐Bosbach et al., 2009). The evaluation of the CSP during observation of action suggests a specific inhibition of the motor system associated with self‐representation. Cortical suppression for actions linked to the self might prevent inappropriate perseveration within the motor system (Schütz‐Bosbach et al., 2009).

A recent study also demonstrated that the RHI paradigm may represent an useful therapeutic approach in improving tactile sensation and rTMS techniques could modulate these effects in healthy subjects and patients with SCI (Frey et al., 2020).

An interesting ERP study revealed that the temporal contiguity of seen and felt tactile stimulation modulates subsequent somatosensory processing at early sensory‐specific stages (Press et al., 2008). These effects seem to be independent of pre‐existing body representations and may affect later stages in the processing of tactile events. Therefore, two types of visuotactile integration process contribute to the “bodily self.” Associative learning based on the temporal contiguity between visual and tactile stimulation enhances perceptual processing of subsequent tactile events; expectations based on the pre‐existing body representations may affect the rate of associative learning that modulates postperceptual, presumably attentional, processing of tactile stimuli.

Evoked potentials studies revealed interactions within the occipital‐premotor network during the RHI, which may reflect hierarchical information exchange. These results are consistent with predictive coding models of multisensory integration and may reflect the attenuation of somatosensory precision that is required to resolve perceptual hypotheses about conflicting multisensory input (Zeller et al., 2015, 2016).

While the temporal time resolution of fMRI or PET is insufficient in distinguishing whether multimodal integration is most directly related to early sensory or later cognitive stages of information processing, EEG and magnetoencephalography (MEG) offer superior temporal resolution for investigating this issue. EEG studies revealed that high‐frequency oscillations play a significant role in the integrative processing of stimuli across modalities (Kanayana et al., 2007, 2009). On the other hand, the γ band activity in the parietal area reflects the visuotactile integration process and that its synchrony causes the illusory intensity (Kanayama et al., 2009).

Other EEG findings provide strong behavioral and neurophysiological evidence of “motor back projection,” in which the movement of an illusory embodied body part is inversely transferred to the sensorimotor system of the observer (Shibuya et al., 2018).

Electroencephalography also revealed spectral dissociations between somatic and visual effects, and higher γ connectivity along the dorsal visual pathways when the rubber hand was embodied (Faivre et al., 2017). In fact, it has been demonstrated that visual percepts are not only influenced by bodily context but are self‐grounded; in the Taylor illusion, the size of an afterimage projected on one's hand changes according to tactile and proprioceptive signals conveying hand position. Therefore, the perception of afterimages depends not only on bodily signals, but also on the sense of self.

During a visuotactile integration task involving a rubber hand, cortical oscillatory components in the high‐frequency (γ) band were detected in the left PC, and the power values of these oscillations were positively correlated with serum BDNF levels. These results suggest that serum BDNF could play a role in modulating the cortical activity in response to visuotactile integration processes related to body image alteration in humans.

Interestingly, patients with schizophrenia exhibit a greater susceptibility to the RHI (Asai et al., 2011; Peled et al., 2003; Thakkar et al., 2011), either because of their specific deficit in predicting the consequences of their voluntary actions (Voss et al., 2010) or because of their altered sense of agency (Daprati et al., 1997; Garbarini et al., 2016; Maeda et al., 2012).

In particular, schizophrenia patients had significant alterations in long‐latency somatosensory evoked responses during the RHI (Peled et al., 2003).

These findings support the hypothesis that associative higher‐level neuronal activity is abnormal in schizophrenia and suggest that the underlying neuropathology of schizophrenia involves higher‐level processing.

## 7 CONCLUSION

The findings of the reviewed neurophysiological studies dealing with the RHI phenomenon provide insight into the functional anatomy of multisensory integration and the perception of oneself and shed new light on our understanding of the different aspects that contribute to the formation of a coherent self‐awareness.

## CONFLICT OF INTEREST

None of the authors have potential conflicts of interest to be disclosed.

## AUTHORS CONTRIBUTION

KS, LuS, LeS, and VV contributed to literature search, data extraction, and data analysis. FV, SG, FT, and RN contributed to the project design and paper writing.

## References

[brb32124-bib-0002] Asai, T., Mao, Z., Sugimori, E., & Tanno, Y. (2011). Rubber hand illusion, empathy, and schizotypal experiences in terms of self‐other representations. Consciousness and Cognition, 20(4), 1744–1750. 10.1016/j.concog.2011.02.005 21371911

[brb32124-bib-0003] Avanzino, L., Bassolino, M., Pozzo, T., & Bove, M. (2011). Use‐dependent hemispheric balance. Journal of Neuroscience, 31(9), 423–3428. 10.1523/JNEUROSCI.4893-10.2011 PMC6623942 21368053

[brb32124-bib-0004] Botvinick, M., & Cohen, J. (1998). Rubber hands ‘feel’ touch that eyes see. Nature, 391(6669), 756. 10.1038/35784 9486643

[brb32124-bib-0005] Burin, D., Garbarini, F., Bruno, V., Fossataro, C., Destefanis, C., Berti, A., & Pia, L. (2017). Movements and body ownership: Evidence from the rubber hand illusion after mechanical limb immobilization. Neuropsychologia, 107, 41–47. 10.1016/j.neuropsychologia.2017.11.004 29109038

[brb32124-bib-0006] Burin, D., Livelli, A., Garbarini, F., Fossataro, C., Folegatti, A., Gindri, P., & Pia, L. (2015). Are movements necessary for the sense of body ownership? Evidence from the rubber hand illusion in pure hemiplegic patients. Public Library of Science ONE, 10(3), e0117155. 10.1371/journal.pone.0117155 25775041 PMC4361688

[brb32124-bib-0007] Daprati, E., Franck, N., Georgieff, N., Proust, J., Pacherie, E., Dalery, J., & Jeannerod, M. (1997). Looking for the agent: An investigation into consciousness of action and self‐consciousness in schizophrenic patients. Cognition, 65(1), 71–86. 10.1016/S0010-0277(97)00039-5 9455171

[brb32124-bib-0008] Della Gatta, F., Garbarini, F., Puglisi, G., Leonetti, A., Berti, A., & Borroni, P. (2016). Decreased motor cortex excitability mirrors own hand disembodiment during the rubber hand illusion. Elife, 20, 5. 10.7554/eLife.14972 PMC5072839 27760692

[brb32124-bib-0009] Di Lazzaro, V., Pilato, F., Saturno, E., Oliviero, A., Dileone, M., Mazzone, P., Insola, A., Tonali, P. A., Ranieri, F., Huang, Y. Z., & Rothwell, J. C. (2005). Theta‐burst repetitive transcranial magnetic stimulation suppresses specific excitatory circuits in the human motor cortex. Journal of Physiology, 565(Pt 3), 945–950. 10.1113/jphysiol.2005.087288 PMC1464561 15845575

[brb32124-bib-0010] Faivre, N., Dönz, J., Scandola, M., Dhanis, H., Bello Ruiz, J., Bernasconi, F., Salomon, R., & Blanke, O. (2017). Self‐grounded vision: Hand ownership modulates visual location through cortical β and γ oscillations. Journal of Neuroscience, 37(1), 11–22. 28053026 10.1523/JNEUROSCI.0563-16.2016 PMC6705670

[brb32124-bib-0011] Fiorio, M., Weise, D., Önal‐Hartmann, C., Zeller, D., Tinazzi, M., & Classen, J. (2011). Impairment of the rubber hand illusion in focal hand dystonia. Brain, 34, 1428–1437. 10.1093/brain/awr026 21378099

[brb32124-bib-0012] Fitzgerald, P. B., Fountain, S., & Daskalakis, Z. J. (2006). A comprehensive review of the effects of rTMS on motor excitability and inhibition. Clinical Neurophysiology, 117(12), 2584–2596. 16890483 10.1016/j.clinph.2006.06.712

[brb32124-bib-0013] Fossataro, C., Bruno, V., Giurgola, S., Bolognini, N., & Garbarini, F. (2018). Losing my hand. Body ownership attenuation after virtual lesion of the primary motor cortex. European Journal of Neuroscience, 48(6), 2272–2287. 10.1111/ejn.14116 30117217

[brb32124-bib-0014] Frey, V. N., Butz, K., Zimmermann, G., Kunz, A., Höller, Y., Golaszewski, S., Trinka, E., & Nardone, R. (2020). Effects of rubber hand illusion and excitatory theta burst stimulation on tactile sensation: A pilot study. Neural Plasticity, 2020, 3069639. 32318103 10.1155/2020/3069639 PMC7152971

[brb32124-bib-0015] Garbarini, F., Mastropasqua, A., Sigaudo, M., Rabuffetti, M., Piedimonte, A., Pia, L., & Rocca, P. (2016). Abnormal sense of agency in patients with schizophrenia: Evidence from bimanual coupling paradigm. Frontiers in Behavioral Neuroscience, 10, e14972. 10.3389/fnbeh.2016.00043 PMC4783405 27014005

[brb32124-bib-0016] Hiramoto, R., Kanayama, N., Nakao, T., Matsumoto, T., Konishi, H., Sakurai, S., Okada, G., Okamoto, Y., & Yamawaki, S. (2017). SBDNF as possible modulator of EEG oscillatory response at the parietal cortex during visuo‐tactile integration processes using a rubber hand. Neuroscience Research, 124, 16–24. 28668502 10.1016/j.neures.2017.05.006

[brb32124-bib-0017] Huang, Y. Z., Edwards, M. J., Rounis, E., Bhatia, K. P., & Rothwell, J. C. (2005). Theta burst stimulation of the human motor cortex. Neuron, 45(2), 201–206. 10.1016/j.neuron.2004.12.033 15664172

[brb32124-bib-0018] Isayama, R., Vesia, M., Jegatheeswaran, G., Elahi, B., Gunraj, C. A., Cardinali, L., Farnè, A., & Chen, R. (2019). Rubber hand illusion modulates the influences of somatosensory and parietal inputs to the motor cortex. Journal of Neurophysiology, 121(2), 563–573. 10.1152/jn.00345.2018 30625001

[brb32124-bib-0019] Kalckert, A., & Ehrsson, H. H. (2012). Moving a rubber hand that feels like your own: A dissociation of ownership and agency. Frontiers in Human Neuroscience, 6, 40. 10.3389/fnhum.2012.00040 22435056 PMC3303087

[brb32124-bib-0020] Kammers, M. P., Verhagen, L., Dijkerman, H. C., Hogendoorn, H., De Vignemont, F., & Schutter, D. J. (2009). Is this hand for real? Attenuation of the rubber hand illusion by transcranial magnetic stimulation over the inferior parietal lobule. Journal of Cognitive Neuroscience, 21(7), 1311–1320. 10.1162/jocn.2009.21095 18752397

[brb32124-bib-0021] Kanayama, N., Morandi, A., Hiraki, K., & Pavani, F. (2017). Causal dynamics of scalp electroencephalography oscillation during the rubber hand illusion. Brain Topography, 30(1), 122–135. 10.1007/s10548-016-0519-x 27620801

[brb32124-bib-0022] Kanayama, N., Sato, A., & Ohira, H. (2007). Crossmodal effect with rubber hand illusion and gamma‐band activity. Psychophysiology, 44(3), 392–402. 10.1111/j.1469-8986.2007.00511.x 17371495

[brb32124-bib-0023] Kanayama, N., Sato, A., & Ohira, H. (2009). The role of gamma band oscillations and synchrony on rubber hand illusion and crossmodal integration. Brain Cognition, 69(1), 19–29. 10.1016/j.bandc.2008.05.001 18555572

[brb32124-bib-0024] Karabanov, A. N., Ritterband‐Rosenbaum, A., Christensen, M. S., Siebner, H. R., & Nielsen, J. B. (2017). Modulation of fronto‐parietal connections during the rubber hand illusion. European Journal of Neuroscience, 45(7), 964–974. 10.1111/ejn.13538 28186673

[brb32124-bib-0025] Lefaucheur, J. P. (2019). Transcranial magnetic stimulation. Handbook of Clinical Neurology, 160, 559–580. 31277876 10.1016/B978-0-444-64032-1.00037-0

[brb32124-bib-0026] Longo, M. R., Schüür, F., Kammers, M. P. M., Tsakiris, M., & Haggard, P. (2008). What is embodiment? A psychometric approach. Cognition, 107(3), 978–998. 10.1016/j.cognition.2007.12.004 18262508

[brb32124-bib-0027] Maeda, T., Kato, M., Muramatsu, T., Iwashita, S., Mimura, M., & Kashima, H. (2012). Aberrant sense of agency in patients with schizophrenia: Forward and backward over‐attribution of temporal causality during intentional action. Psychiatry Research, 198, 1–6. 10.1016/j.psychres.2011.10.021 22374553

[brb32124-bib-0028] Mioli, A., D'Alonzo, M., Pellegrino, G., Formica, D., & Di Pino, G. (2018). Intermittent theta burst stimulation over ventral premotor cortex or inferior parietal lobule does not enhance the rubber hand illusion. Frontiers of Neuroscience, 23(12), 870. 10.3389/fnins.2018.00870 PMC6265367 30532689

[brb32124-bib-0046] Paulus, W., Classen, J., Cohen, L.G., Large, C.H., Di Lazzaro, V., Nietsche, M., Pascual‐Leone, A., Rosenow, F., Rothwell, J. C., & Ziemann, U. (2008). State of the art: pharmacologic effects on cortical excitability measures tested by transcranial magnetic stimulation. Brain Stimul, 1, 151–163. 20633382 10.1016/j.brs.2008.06.002

[brb32124-bib-0029] Peled, A., Pressman, A., Geva, A. B., & Modai, I. (2003). Somatosensory evoked potentials during a rubber‐hand illusion in schizophrenia. Schizophrenia Research, 64(2–3), 157–163. 10.1016/S0920-9964(03)00057-4 14613680

[brb32124-bib-0030] Peviani, V., Magnani, F. G., Ciricugno, A., Vecchi, T., & Bottini, G. (2018). Rubber hand illusion survives ventral premotor area inhibition: A rTMS study. Neuropsychologia, 120, 18–24. 10.1016/j.neuropsychologia.2018.09.017 30266289

[brb32124-bib-0031] Press, C., Heyes, C., Haggard, P., & Eimer, M. (2008). Visuotactile learning and body representation: An ERP study with rubber hands and rubber objects. Journal of Cognitive Neuroscience, 20(2), 312–323. 10.1162/jocn.2008.20022 18275337 PMC2373573

[brb32124-bib-0032] Rao, I. S., & Kayser, C. (2017). Neurophysiological correlates of the rubber hand illusion in late evoked and alpha/beta band activity. Frontiers of Human Neuroscience, 2017(11), 377. 10.3389/fnhum.2017.00377 PMC5524680 28790906

[brb32124-bib-0033] Scandola, M., Tidoni, E., Avesani, R., Brunelli, G., Aglioti, S. M., & Moro, V. (2014). Rubber hand illusion induced by touching the face ipsilaterally to a deprived hand: Evidence for plastic “somatotopic” remapping in tetraplegics. Frontiers in Human Neuroscience, 8, 404. 24959128 10.3389/fnhum.2014.00404 PMC4050649

[brb32124-bib-0034] Schütz‐Bosbach, S., Avenanti, A., Aglioti, S. M., & Haggard, P. (2009). Don't do it! Cortical inhibition and self‐attribution during action observation. Journal of Cognitive Neuroscience, 21(6), 1215–1227. 10.1162/jocn.2009.21068 18702585

[brb32124-bib-0035] Schütz‐Bosbach, S., Mancini, B., Aglioti, S. M., & Haggard, P. (2006). Self and other in the human motor system. Current Biology, 16(18), 1830–1834. 10.1016/j.cub.2006.07.048 16979561

[brb32124-bib-0036] Shibuya, S., Unenaka, S., Zama, T., Shimada, S., & Ohki, Y. (2018). Spontaneous imitative movements induce by an illusory embodied fake hand. Neuropsychologia, 111, 77–84. 29407592 10.1016/j.neuropsychologia.2018.01.023

[brb32124-bib-0047] Siebner, H. R., & Rothwell, J. C. (2003). Transcranial magnetic stimulation: new insights into representational cortical plasticity. Exp. Brain Res, 148, 1–16. 12478392 10.1007/s00221-002-1234-2

[brb32124-bib-0038] Thakkar, K. N., Nichols, H. S., McIntosh, L. G., & Park, S. (2011). Disturbances in body ownership in schizophrenia: Evidence from the rubber hand illusion and case study of a spontaneous out‐of‐body experience. Public Library of Science ONE, 6(10), e14972. 10.1371/journal.pone.0027089 PMC3205058 22073126

[brb32124-bib-0039] Ticini, L. F., Dolk, T., Waszak, F., & Schütz‐Bosbach, S. (2018). IPL‐M1 interaction shapes pre‐reflective social differentiation in the human action system: New insights from TBS and TMS combined. Scientific Reports, 8(1), 12001. 10.1038/s41598-018-30480-z 30097641 PMC6086836

[brb32124-bib-0040] Tidoni, E., Grisoni, L., Liuzza, M. T., & Aglioti, S. M. (2014). Rubber hand illusion highlights massive visual capture and sensorimotor face‐hand remapping in a tetraplegic man. Restorative Neurology and Neuroscience, 32(5), 611–622. 10.3233/RNN-130385 25015700

[brb32124-bib-0041] Voss, M., Moore, J., Hauser, M., Gallinat, J., Heinz, A., & Haggard, P. (2010). Altered awareness of action in schizophrenia: A specific deficit in predicting action consequences. Brain, 133(10), 3104–3112. 10.1093/brain/awq152 20685805

[brb32124-bib-0042] Wittenberg, G. F., & Schaechter, J. D. (2009). The neural basis of constraint‐induced movement therapy. Current Opinion in Neurology, 22(6), 582–588. 10.1097/WCO.0b013e3283320229 19741529

[brb32124-bib-0043] Wold, A., Limanowski, J., Walter, H., & Blankenburg, F. (2014). Proprioceptive drift in the rubber hand illusion is intensified following 1 Hz TMS of the left EBA. Frontiers in Human Neuroscience, 8, 390. 10.3389/fnhum.2014.00390 24926247 PMC4045244

[brb32124-bib-0044] Zeller, D., Friston, K. J., & Classen, J. (2016). Dynamic causal modeling of touch‐evoked potentials in the rubber hand illusion. NeuroImage, 38, 266–273. 10.1016/j.neuroimage.2016.05.065 27241481

[brb32124-bib-0045] Zeller, D., Litvak, V., Friston, K. J., & Classen, J. (2015). Sensory processing and the rubber hand illusion – An evoked potentials study. Journal of Cognitive Neuroscience, 27(3), 573–582. 10.1162/jocn_a_00705 25170795

